# Problematic Internet Use Before and During the COVID-19 Pandemic in Youth in Outpatient Mental Health Treatment: App-Based Ecological Momentary Assessment Study

**DOI:** 10.2196/33114

**Published:** 2022-01-28

**Authors:** Meredith Gansner, Melanie Nisenson, Vanessa Lin, Sovannarath Pong, John Torous, Nicholas Carson

**Affiliations:** 1 Department of Psychiatry Cambridge Health Alliance Cambridge, MA United States; 2 Department of Digital Psychiatry Beth Israel Deaconess Medical Center Boston, MA United States

**Keywords:** COVID-19, problematic internet use, ecological momentary assessment, internet, app, youth, young adult, teenager, outpatient, mental health, treatment, pilot, cohort, change

## Abstract

**Background:**

Youth with existing psychiatric illness are more apt to use the internet as a coping skill. Because many “in-person” coping skills were not easily accessible during the COVID-19 pandemic, youth in outpatient mental health treatment may have been particularly vulnerable to the development of problematic internet use (PIU). The identification of a pandemic-associated worsening of PIU in this population is critical in order to guide clinical care; if these youth have become dependent upon the internet to regulate their negative emotions, PIU must be addressed as part of mental health treatment. However, many existing studies of youth digital media use in the pandemic do not include youth in psychiatric treatment or are reliant upon cross-sectional methodology and self-report measures of digital media use.

**Objective:**

This is a retrospective cohort study that used data collected from an app-based ecological momentary assessment protocol to examine potential pandemic-associated changes in digital media youth in outpatient mental health treatment. Secondary analyses assessed for differences in digital media use dependent upon personal and familial COVID-19 exposure and familial hospitalization, as well as factors associated with PIU in this population.

**Methods:**

The participants were aged 12-23 years and were receiving mental health treatment in an outpatient community hospital setting. All participants completed a 6-week daily ecological momentary assessment protocol on their personal smartphones. Questions were asked about depression (PHQ-8 [8-item Patient Health Questionnaire]), anxiety (GAD-7 [7-item General Anxiety Disorder]), PIU (PIU-SF-6 [Problematic Internet Use Short Form 6]), digital media use based on Apple’s daily screen time reports, and personal and familial COVID-19 exposure. The analyses compared screen time, psychiatric symptoms, and PIU between cohorts, as well as between youth with personal or familial COVID-19 exposures and those without. The analyses also assessed for demographic and psychiatric factors associated with clinically significant PIU-SF-6 scores.

**Results:**

A total of 69 participants completed the study. The participants recruited during the pandemic were significantly more likely to meet the criteria for PIU based on their average PIU-SF-6 score (*P*=.02) and to spend more time using social media each day (*P*=.049). The overall amount of daily screen time did not differ between cohorts. Secondary analyses revealed a significant increase in average daily screen time among subjects who were exposed to COVID-19 (*P*=.01). Youth with clinically significant PIU-SF-6 scores were younger and more likely to have higher PHQ-8 (*P*=.003) and GAD-7 (*P*=.003) scores. No differences in scale scores or media use were found between subjects based on familial COVID-19 exposure or hospitalization.

**Conclusions:**

Our findings support our hypothesis that PIU may have worsened for youth in mental health treatment during the pandemic, particularly the problematic use of social media. Mental health clinicians should incorporate screening for PIU into routine clinical care in order to prevent potential familial conflict and subsequent psychiatric crises that might stem from unrecognized PIU.

## Introduction

Significant concerns exist that youth mental health worsened during the COVID-19 pandemic. While the US emergency department visits for pediatric ailments such as asthma or otitis media decreased during the pandemic, the proportion of youth presentations related to mental health crises increased in 2020 [1]. Specifically, more recent national data show that emergency department visits for suicidal ideation increased for youth aged 12-17 years, especially for adolescent girls [2]. International data also support these concerns, including a meta-analysis of 29 studies that demonstrated increased levels of youth anxiety and depression during the pandemic [3].

Researchers leading these studies have been careful to note that their study designs do not allow for the determination of causality. The pandemic has not been proven to be the direct cause of worsening psychiatric illness, despite growing evidence that pandemic restrictions likely had a significant impact on youth mental health. News outlets have featured stories from youth explicitly stating that pandemic-associated stressors such as online schooling and cancelled extracurricular activities led to a worsening of anxiety or depression [4]. Moreover, access to some mental health services, such as those available through schools or other community-based supports, also became limited during the early months of the pandemic [5]. The compounding of these 2 factors may have created an opportune environment for an additional influencer of youth mental health, that of problematic screen time.

Elevated daily screen time and problematic internet use (PIU), an excessive, uncontrollable drive to continue use of the internet despite negative consequences, are both well-associated with numerous psychiatric comorbidities, including depression, anxiety, substance use, self-injurious behavior, and suicidality [6-9]. While increased levels of screen time were a recognized consequence of the pandemic for individuals across the developmental lifespan, youth are the largest consumers of digital media and the most likely population to develop PIU. Thus, it is potentially unsurprising that emerging studies have identified a comorbid increase in youth screen time and severity of psychiatric symptoms during the pandemic [10,11].

However, not all youth appear equally susceptible to PIU and the negative effects of screen time. Youth with existing psychiatric illness, for example, may be especially vulnerable to PIU; our prior longitudinal studies in this specific population have highlighted momentary negative correlations between cell phone engagement, PIU, and mood symptom severity, suggesting that these youth use digital media to relieve psychiatric symptoms, subsequently risking PIU development [12,13]. Therefore, youth in mental health treatment may have developed a particularly complicated relationship with digital media as a result of the COVID-19 pandemic.

This study assessed the digital media habits of 2 separate cohorts of youth (1 before and 1 during the COVID-19 pandemic) who were receiving mental health treatment in a single community health setting. Data were obtained from an existing ecological momentary assessment (EMA) smartphone protocol that collected daily information about a participant’s qualitative digital media use, PIU, and symptoms of anxiety and depression over a 6-week period. Through the examination of these collected data, our study (1) assessed how digital media use and mental health may have changed for youth in mental health treatment during the pandemic and (2) explored how personal or familial exposure to the novel coronavirus might have impacted digital media habits and mental health. Due to more limited access to nondigital coping skills during the pandemic, we hypothesized that youth in the pandemic cohort would have higher rates of PIU and spend more time on screens and social media. We also hypothesized that within the COVID-19 cohort, youth personally exposed to COVID-19 might have significantly higher amounts of daily screen and social media time due to increased awareness of the disease and subsequent avoidance of in-person pastimes.

The clinical implications of this study are significant. If these high-risk youth developed a more pathological relationship with digital media during the pandemic, they may have a particularly difficult time separating from digital devices when COVID-19 restrictions are eventually rolled back in favor of in-person activities and services. Because forced separation from devices is often a trigger for parent-child conflict and can precipitate a psychiatric crisis [14], mental health professionals need to be aware of this increased risk to their patients and be prepared to help parents and guardians safely facilitate device separation.

## Methods

### Participants

The study participants were initially recruited as part of a separate app-based EMA pilot study investigating PIU in this population [12] and were all patients of outpatient mental health clinics within the network of a large community-based hospital in the greater Boston area. The participants were eligible for this separate EMA study if they were between 12 and 23 years old at the beginning of the study and owned a personal smartphone. If a potential participant was under 18 years of age, an informed consent was obtained from the parent or guardian. The participants were excluded if parental or guardian consent was not obtained (if <18 years old) or if they were unable to read English at a 6th grade level (due to lack of app availability in languages other than English). The pre–COVID-19 cohort was passively recruited at the clinics through posted fliers and actively recruited via referral to the study team from the participant’s mental health care provider. All participants referred from providers assented to the referral. For the COVID-19 sample, the participants were actively recruited by the study team through the hospital’s electronic health record (EHR) system. Notably, study recruitment was paused temporarily the day after the state’s declaration of emergency due to COVID-19 on March 10, 2020, due to the requisite need to switch to remote recruitment methods only. Recruitment began again in September 2020 once the Institutional Review Board approval was granted for remote study recruitment and changes were made in the protocol to include questions about COVID-19 exposure. For these analyses, the participants were retroactively categorized into pre–COVID-19 and COVID-19 cohorts based on whether they were recruited before or after the halt in recruitment on March 11, 2020. The participants were compensated with a $25 Amazon gift card at the beginning and end of the study period. Compensation was not dependent on the level of engagement with the app. All parts of this study were reviewed and approved by the hospital’s Institutional Review Board and conformed to the latest version of the Declaration of Helsinki.

### Procedure

Data for this study had previously been collected by these authors as part of a separate app-based EMA pilot protocol that used mindLAMP for daily assessment and data collection over a period of 6 weeks. MindLAMP is a free-rein research platform that includes both an online portal system and a smartphone app [15]. All study participants downloaded the mindLAMP app onto their smartphones prior to the start of their study period. The participants were reminded to complete daily surveys via a push notification sent via the app. A time of day was selected so that the participants would likely be at home, allowing for more privacy to complete the surveys.

The surveys included 3 clinical scales to measure PIU (PIU-SF-6 [Problematic Internet Use Short Form 6]), depression (PHQ-8 [8-item Patient Health Questionnaire]), and anxiety (GAD-7 [7-item General Anxiety Disorder]). The PHQ-8 is a modified version of the PHQ-9, which omits the final question assessing suicidality due to the fact that positive responses could not be actively monitored remotely. The PIU-SF-6 is a scale validated for the measurement of PIU in youth (=.77) [16]. Wording of the 3 scales was also adjusted to account for their being administered on a daily basis. It does not appear that daily administration impacts scale validity [17]. Each participant who owned an iPhone was asked to input the following information provided daily by the Apple screen time report feature of iOS: total screen time, total time on social media, and top 3 apps used that day. As part of the screen time feature, daily time spent on social media is automatically identified, categorized, and calculated. Media that are considered social media include both website browser and app visits to sites such as Facebook, WhatsApp, Instagram, or Apple Messenger. Screen time reporting for Android was not available at the time the initial study protocol was approved; however, the majority of the study participants had iPhones. Each time the participant connected to Wi-Fi, mindLAMP uploaded de-identified survey data to a secure server compliant with the Health Insurance Portability and Accountability Act of 1996.

Participant psychiatric diagnoses were obtained from the most recent mental health visit notes in the participant’s EHR. Youth completing the study during the COVID-19 pandemic were asked to respond to a brief survey regarding their personal and familial exposure to the novel coronavirus at the beginning and end of the study period. For the purpose of this study, family was defined as whomever the youth considered to be family, not just those individuals living with the participant.

### Processing and Analysis

Data were downloaded from mindLAMP in the form of daily scale scores, screen and social media time, and the 3 most commonly used apps on a daily basis. For each participant, daily PIU-SF-6, PHQ-8, and GAD-7 scale scores were transformed into average scale scores. This average scale score was then used to create a secondary binary variable, which described if a participant’s average score met or exceeded standardized cut-off values when screening for clinical illness. For the PIU-SF-6, this threshold was a score of ≥15 [16], and ≥10 for the PHQ-8 and GAD-7 scales. Gender was defined as the current gender identity at study enrollment. Participant diagnoses obtained from the EHR were transformed into 2 binary variables: the presence of an anxiety disorder (“yes” or “no”) and the presence of a depressive disorder (“yes” or “no”) to compare preexisting diagnoses across samples. Average daily times (in minutes) spent using a smartphone or social media were calculated for each participant.

Addressing the study’s first goal, logistic regressions compared the number of participants whose average scale scores met clinical cut-off values for the PHQ-8, GAD-7, and PIU-SF-6 across pre–COVID-19 and COVID-19 cohorts. While the sample size of the study is a notable limitation for this model, logistic regression models were chosen for this analysis in order to adjust for confounders of age and gender. Age is a known confounder positively associated with both youth screen time and social media use; therefore, the significant difference in age between pre–COVID-19 and COVID-19 cohorts needed to be considered in this first analysis. Due to the nonnormality of the data, Mann-Whitney tests were performed to assess for differences in mean daily screen and social media times across these groups. To correct for age differences when comparing screen and social media times across cohorts, these specific data underwent intercept adjustment for age and gender prior to conducting Mann-Whitney tests.

A second set of analyses assessed for differences in screen and social media times as well as average scale scores based on COVID-19 exposure within the COVID-19 cohort alone. These analyses used the Fisher exact and Mann-Whitney tests due to the small sample size. Given the unique stress of the pandemic upon minority populations, we also assessed whether youth of color in the COVID-19 cohort had significantly different digital media use compared with nonminority youth.

Finally, we sought to characterize our sample with PIU comparing participants with average PIU-SF-6 scores meeting the clinical cut-off score of ≥15 to those with scores of <15. We assessed associations between age, gender, minority status, and likelihood of having clinically significant GAD-7 and PHQ-8 scores and preexisting anxiety or depressive disorder diagnoses using the Fisher exact tests. As mentioned, Mann-Whitney tests assessed for differences in age and average daily screen or social media time. All analyses were performed using Stata (version 1.2.5033, Stata Corp) [18] and Rstudio (version 1.2.5033, RStudio Inc) [19].

## Results

A total of 69 participants completed the 6-week study. Symptom scale data were obtained from all 69 participants, and 77% (n=53) of the participants had iPhones and provided information about their smartphone use from daily screen time reports. Demographic information is summarized in Table 1. While the participants in the COVID-19 sample were significantly older than youth in the pre–COVID-19 sample, there were no differences in gender or prevalence of preexisting anxiety or depressive disorders between the groups.

**Table 1:** Participant demographic information.

Characteristics			Values				
			Pre–COVID-19		COVID-19		*P* value
Total			27		42		N/A^a^
Age (years), mean (SD)			15.30 (2.74)		16.95 (1.94)		.003
**Gender, n (%)**							.07
	Male	13 (48.1)		10 (23.8)			
	Female	14 (51.9)		32 (76.2)			
**Race, n (%)**							.24
	White	14 (51.9)		21 (50.0)			
	Hispanic or Latinx	7 (25.9)		10 (23.8)			
	Black	5 (18.5)		10 (23.8)			
	Asian	1 (3.7)		1 (2.4)			

^a^N/A: not applicable.

**Figure 1:**
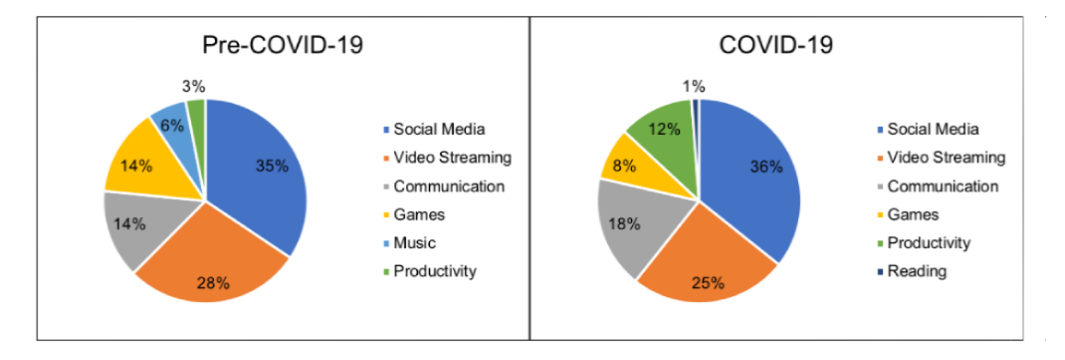
Most frequently used types of apps based on Apple screen time reports.

Controlling for age and gender, youth in our COVID-19 cohort were more likely to meet criteria for PIU based on their PIU-SF-6 scores averaged over the 6-week study (*P*=.02) (Table 2). The averaged PHQ-8 and GAD-7 scale scores were also higher in the COVID-19 cohort, but these increases did not reach statistical significance. Social media apps were the most popular type of app used both prior to and during the pandemic (Figure 1); however, again controlling for age and gender, the amount of time spent daily on social media was significantly higher in those youth who completed the study during the pandemic (*P*=.049).

**Table 2:** Comparison of daily survey scores between pre–COVID-19 and COVID-19 cohorts.

Surveys	Values			
	Pre–COVID-19	COVID-19	β	*P* value
Average PIU-SF-6^a^ ≥15, n (%)	1 (3.7)	10 (23.8)	2.99	.02
Average PHQ-8^b^ ≥10, n (%)	8 (29.6)	16 (41.0)	.22	.72
Average GAD-7^c^ ≥10, n (%)	2 (7.4)	11 (28.2)	1.44	.11
Average daily screen time, mean minutes (SD)	351.46 (204.32)	380.47 (135.23)	—^d^	.10
Average daily social media time, mean minutes (SD)	123.14 (77.58)	173.23 (84.80)	—	.049

^a^PIU-SF-6: Problematic Internet Use Short Form 6.

^b^PHQ-8: 8-item Patient Health Questionnaire.

^c^GAD-7: 7-item General Anxiety Disorder.

^d^Not available.

Of those participants in the COVID-19 cohort, youth with a personal history of COVID-19 exposure reported a significantly higher average daily screen time (*P*=.01) (Table 3). However, familial COVID-19 diagnoses and hospitalizations did not appear to be related to changes in digital media use or higher daily PIU-SF-6, PHQ-8, or GAD-7 scores. There were no significant differences between age and gender between youth with personal or family exposure to COVID-19 and those without. Of those participants who reported COVID-19 exposure, the majority (67% [n=4]) were youth of color. By contrast, among participants without a history of COVID-19 exposure, only 47% (n=17) identified as youth of color. Youth of color in the COVID-19 pandemic did not have significantly higher rates of PIU, screen time, or social media time (*P*=.72, *P*=.12, and *P*=.45, respectively). Overall, youth with PIU-SF-6 scores of ≥15 in our study population were significantly younger and more likely to have comorbid clinically elevated PHQ-8 and GAD-7 scores (*P*=.003) (Table 4).

**Table 3:** Associations between personal and familial COVID-19 exposure, psychiatric symptoms, and daily smartphone use.

Averaged surveys for infections and hospitalization			Values					
			No		Yes		*P* value	
**Personal COVID-19 infection**								
	Total	36		6		N/A^a^		
	Average PIU-SF-6^b^ ≥15, n (%)	9 (25.0)		1 (16.7)		.99		
	Average PHQ-8^c^ ≥10, n (%)	13 (38.2)		3 (60.0)		.63		
	Average GAD-7^d^ ≥10, n (%)	9 (26.5)		2 (40.0)		.61		
	Average daily screen time, mean minutes (SD)	356 (126)		548 (54)		.01		
	Average daily social media time, mean minutes (SD)	166 (85)		221 (76)		.26		
**Familial COVID-19 infection**								
	Total	14		23		N/A		
	Average PIU-SF-6 ≥15, n (%)	3 (15.8)		7 (30.4)		.31		
	Average PHQ-8 ≥10, n (%)	6 (35.3)		10 (45.5)		.74		
	Average GAD-7 ≥10, n (%)	5 (29.4)		6 (27.3)		.99		
	Average daily screen time, mean minutes (SD)	365 (102)		391 (156)		.55		
	Average daily social media time, mean minutes (SD)	143 (72)		192 (89)		.16		
**Familial COVID-19 hospitalization**								
	Total	27		10		N/A		
	Average PIU-SF-6 ≥15, n (%)	7 (21.9)		3 (30)		.68		
	Average PHQ-8 ≥10, n (%)	11 (37.9)		5 (50)		.71		
	Average GAD-7 ≥10, n (%)	9 (31)		2 (20)		.69		
	Average daily screen time, mean minutes (SD)	356 (136)		443 (119)		.11		
	Average daily social media time, mean minutes (SD)	161 (89)		204 (68)		.07		

^a^N/A: not applicable.

^b^PIU-SF-6: Problematic Internet Use Short Form 6.

^c^PHQ-8: 8-item Patient Health Questionnaire.

^d^GAD-7: 7-item General Anxiety Disorder.

**Table 4:** Participant comparisons based on average Problematic Internet Use Short Form 6 scores.

Characteristics	Average PIU-SF-6^a^ <15	Average PIU-SF-6 ≥15	*P* value
Age (years), mean (SD)	16.4 (2.5)	15.7 (1.8)	.27
Female/male, n (%)	37/21 (63.8/36.2)	9/2 (81.8/18.2)	.31
Minority youth, n (%)	28 (48.3)	6 (54.5)	.75
Preexisting anxiety disorder diagnosis, n (%)	36 (62.1)	6 (54.5)	.74
Preexisting depressive disorder diagnosis, n (%)	38 (65.5)	7 (63.6)	.99
Average PHQ-8^b^ ≥10, n (%)	16 (28.6)	8 (80.0)	.003
Average GAD-7^c^ ≥10, n (%)	7 (12.5)	6 (60.0)	.003
Average daily screen time, mean (SD)	359.2 (166.5)	424.1 (152.8)	.12
Average daily social media time, mean (SD)	146.5 (83.1)	188.8 (91.3)	.19

^a^PIU-SF-6: Problematic Internet Use Short Form 6.

^b^PHQ-8: 8-item Patient Health Questionnaire.

^c^GAD-7: 7-item General Anxiety Disorder.

## Discussion

Our results demonstrate that the COVID-19 pandemic may have altered digital media habits in youth with psychiatric illness. Study participants assessed over 6 weeks during the pandemic were significantly more likely to endorse consistent feelings of problematic dependency on the internet. Because rates of preexisting anxiety and depressive disorders did not differ significantly between cohorts, the higher rates of PIU seen in the COVID-19 cohort were likely not attributable to the participants’ preexisting psychiatric disorders. However, prior studies have consistently emphasized positive correlations between PIU and active psychiatric symptoms, including in youth in mental health treatment [7-9,12]. This connection between active psychiatric distress and PIU is also supported by our study’s finding that participants with PIU were more likely to report experiencing clinically significant symptoms of anxiety and depression during the study period. Thus, in the presence of active psychiatric symptoms, youth in our study population may be predisposed to develop PIU in environments of increased stress, such as a pandemic.

Some studies have suggested that excessive internet use and PIU directly cause adverse mental health outcomes [20]. However, our previous pilot studies using ecological momentary assessment and digital phenotyping have shown that for youth in mental health treatment, screen time and PIU are linked to temporary improvements in anxiety and depressive symptoms [12,13]. Existing research indicates that youth with mental health difficulties are more inclined to turn to online peer support to manage health issues; for example, youth with moderate-to-severe depressive symptoms are more likely to seek out peers’ health-related stories posted online [21]. Because in-person supports were more challenging to access during the pandemic, especially mental health services offered through school or in-home visits, youth in our study population may have gone online to help regulate their negative emotions. This hypothesis is further reinforced by our finding that youth with psychiatric diagnoses in the COVID-19 cohort spent a significantly larger percentage of their daily screen time on social media. Social media platforms specifically can offer interpersonal connection and external validation, opportunities for which were more limited during the pandemic. Thus, without their usual mental health treatments or coping skills consistently available, these youth may have been at particularly high risk of developing or reinforcing habitual reliance upon social media as a primary coping skill.

The fact that average screen time did not also increase during the pandemic in our study population may reflect our participants’ high baseline rates of digital media use compared with youth without active psychiatric symptoms [21,22]. However, in the COVID-19 cohort, youth who reported a history of COVID-19 exposure used their smartphones significantly more on a daily basis; these youths’ iPhone screen time summaries indicated 54% more minutes of daily screen time than participants without such history. Notably, all participants with a known history of COVID-19 exposure were exposed before their 6-week study period, and only 50% of exposed participants subsequently contracted the virus, suggesting that illness and requisite quarantine were unlikely to be the sole contributors to this increase in phone use. We hypothesize that youth exposed to COVID-19 may have been more likely to appreciate the risks associated with the virus and therefore relied on virtual rather than in-person pastimes out of fear of contracting the virus. Additionally, the majority of our participants exposed to COVID-19 were youth of color, compared to youth who were not exposed, where the majority were White and non-Hispanic/Latinx. It has been well established that ethnic and racial minority communities are at greater risk of COVID-19 due to systemic racism impacting health care access, housing, and occupation [23], and communities with higher rates of COVID-19 transmission may have been particularly limited in the ability to provide in-person mental health services or safe spaces for in-person interactions. Moreover, many adults in these communities were essential workers and unable to stay home with children to monitor and provide guidance surrounding the amount of daily screen time. As youth of color did not have higher rates of screen time or PIU during the pandemic, the combination of multiple psychosocial stressors and COVID-19 exposure may have been necessary for triggering increased smartphone engagement.

These findings have significant implications for the treatment of youth with psychiatric diagnoses. While it is always important for clinicians to revisit a patient’s digital media habits periodically throughout the course of treatment, the pandemic may necessitate additional screening for changes in media use. Youth struggling with their psychiatric symptoms or with a history of COVID-19 exposure may also benefit from PIU screening specifically, and their parents or guardians should be asked about conflicts arising surrounding separation from devices, particularly smartphones. A positive screen will allow for the careful development of a thoughtful, gradated media plan to help youth move back into healthier patterns of digital media use and begin intentional practice of coping skills that are independent of screens. Ideally, these youth will be more successful re-adjusting to aspects of screen-free daily living if the transition is predictable and gradual and involves youth input.

Finally, social media research in this population is challenging; even in adults, the recall accuracy of daily screen time is limited [24], and the finding that many younger populations use digital media continuously [25] likely further impacts recall accuracy. This study’s use of EMA data afforded us a better opportunity to appreciate ecologically valid and objective changes in youth digital media use through longitudinal sampling and procurement of Apple screen time summaries. By asking our participants to provide us with their daily screen time reports, we were able to gather both qualitative and quantitative data regarding smartphone use in a population subset where protocol adherence can be challenging. Assessing the feasibility of app-based EMA as a clinical intervention was not the primary goal of this study. However, monthly visits are standard of care in pediatric psychiatry, and the majority of our participants provided psychiatric symptom updates on at least a weekly basis; therefore, there may be a clinical role for app-based EMA in this population, particularly to track changes in digital media use and associated mood symptoms.

Our research findings cannot conclude that the pandemic was the root cause of worsening youth mental health or PIU, and the study’s small sample size is a notable limitation. Our data suggest that youth in mental health treatment were at increased risk of PIU development during the pandemic, specifically those with more severe symptoms of anxiety and depression. Moreover, those youth in mental health treatment exposed to COVID-19 endorsed greater amounts of daily smartphone use than those without a history of exposure. Based on our results, we recommend that clinicians screen high-risk pediatric patients for potential pandemic-associated changes in digital media habits as this may prevent psychiatric crises secondary to digital media-related conflict in the home or at school. From a systems standpoint, such crisis prevention measures may ease the burdens placed on our already overwhelmed psychiatric crisis teams and emergency rooms as we continue to navigate the COVID-19 pandemic.

## Abbreviations

EHR: electronic health record
EMA: ecological momentary assessment
GAD-7: 7-item General Anxiety Disorder
PHQ-8: 8-item Patient Health Questionnaire
PIU: problematic internet use
PIU-SF-6: Problematic Internet Use Short Form 6
